# The Role of CT-Quantified Body Composition on Longitudinal Health-Related Quality of Life in Colorectal Cancer Patients: The Colocare Study

**DOI:** 10.3390/nu12051247

**Published:** 2020-04-28

**Authors:** Biljana Gigic, Johanna Nattenmüller, Martin Schneider, Yakup Kulu, Karen L. Syrjala, Jürgen Böhm, Petra Schrotz-King, Hermann Brenner, Graham A. Colditz, Jane C. Figueiredo, William M. Grady, Christopher I. Li, David Shibata, Erin M. Siegel, Adetunji T. Toriola, Hans-Ulrich Kauczor, Alexis Ulrich, Cornelia M. Ulrich

**Affiliations:** 1 Department of General, Visceral and Transplantation Surgery, Heidelberg University Hospital, 69120 Heidelberg, Germany; 2 Division of Preventive Oncology, National Center for Tumor Diseases and German Cancer Research Center, 69120 Heidelberg, Germany; 3 Department of Diagnostic and Interventional Radiology, Heidelberg University Hospital, 69120 Heidelberg, Germany; 4 Clinical Research Division, Fred Hutchinson Cancer Research Center, Seattle, WA 98109, USA; 5 Population Sciences, Huntsman Cancer Institute, Salt Lake City, UT 84112, USA; 6 Division of Clinical Epidemiology and Aging Research, German Cancer Research Center, 69120 Heidelberg, Germany; 7 German Cancer Consortium (DKTK), German Cancer Research Center, 69120 Heidelberg, Germany; 8 Division of Public Health Sciences, Department of Surgery, Washington University School of Medicine and Siteman Cancer Center, St Louis, MO 63110, USA; 9 Department of Medicine, Samuel Oschin Comprehensive Cancer Institute, Cedars-Sinai Medical Center, Los Angeles, CA 90048, USA; 10 Public Health Sciences Division, Fred Hutchinson Cancer Research Center, Seattle, WA 98109, USA; 11 Department of Medicine, University of Washington School of Medicine, Seattle, WA 98195, USA; 12 Department of Surgery, University of Tennessee Health Science Center, Memphis, TN 38163, USA; 13 Cancer Epidemiology Program, H. Lee Moffitt Cancer Center and Research Institute, Tampa, FL 33612, USA; 14 Department of Population Health Sciences, University of Utah, Salt Lake City, UT 84112, USA

**Keywords:** visceral fat area, subcutaneous fat area, skeletal muscle mass, CT-quantified body composition, health-related quality of life, colorectal cancer, prospective data

## Abstract

Background: Obesity, defined by body mass index (BMI), measured at colorectal cancer (CRC) diagnosis has been associated with postoperative complications and survival outcomes. However, BMI does not allow for a differentiation between fat and muscle mass. Computed tomography (CT)-defined body composition more accurately reflects different types of tissue and their associations with health-related quality of life (HRQoL) during the first year of disease, but this has not been investigated yet. We studied the role of visceral and subcutaneous fat area (VFA and SFA) and skeletal muscle mass (SMM) on longitudinally assessed HRQoL in CRC patients. Methods: A total of 138 newly diagnosed CRC patients underwent CT scans at diagnosis and completed questionnaires prior to and six and twelve months post-surgery. We investigated the associations of VFA, SFA, and SMM with HRQoL at multiple time points. Results: A higher VFA was associated with increased pain six and twelve months post-surgery (β = 0.06, *p* = 0.04 and β = 0.07, *p* = 0.01) and with worse social functioning six months post-surgery (β = −0.08, *p* = 0.01). Higher SMM was associated with increased pain twelve months post-surgery (β = 1.03, *p* < 0.01). Conclusions: CT-quantified body composition is associated with HRQoL scales post-surgery. Intervention strategies targeting a reduction in VFA and maintaining SMM might improve HRQoL in CRC patients during the first year post-surgery.

## 1. Introduction

Colorectal cancer is the second leading cause of cancer deaths worldwide [1]. Besides recurrence and survival, health-related quality of life (HRQoL) is an important clinical outcome in cancer patients [2]. As the number of colorectal cancer survivors grows steadily [3], addressing the causes of low HRQoL is a top priority. Colorectal cancer survivors frequently report the deterioration of physical functions, fatigue or bowel dysfunction due to the disease and its treatment, which can severely impact patients’ HRQoL [2,4,5]. HRQoL, including physiological, psychological, and social wellbeing [2], in colorectal cancer patients is threatened by health problems persisting for years after diagnosis and treatment [6]; thus, there is a strong need for the early identification of patient characteristics that determine patients’ HRQoL and to develop preventive and targeted intervention strategies (e.g., nutritional support or individual exercise training) [7,8]. Recently, a comprehensive literature review by Bours et al. provided an overview of biopsychological factors associated with HRQoL among colorectal cancer patients [6]. Obesity was consistently reported to be associated with worse HRQoL outcomes. Previous research showed that, in a period between six months and ten years after diagnosis, a higher body mass index (BMI) or the presence of obesity was associated with lower HRQoL in colorectal cancer survivors [9,10,11]. However, BMI does not allow for the differentiation between fat and muscle mass. Distinguishing between different body compartments is required, as (for example) muscle mass plays an important role in physical functions, whereas muscle depletion, also termed sarcopenia, is pathophysiologically involved in aging, but also in metabolic syndromes, fatty liver disease, cancer and various chronic diseases with increased mortality [12,13,14,15,16]. Moreover, BMI is insufficient for differentiating between the pathophysiologically important distinction between visceral and subcutaneous fat tissue [17]. Computed tomography (CT) imaging is routinely used as part of the clinical management of cancer patients. CT-defined body composition more accurately reflects different types of adipose tissue as well as muscle mass [18]. Previous studies using CT-based body composition parameters revealed associations between visceral adiposity and increased rates of postoperative complications, longer hospitalization, and impaired disease-free survival [19,20,21,22,23]. Moreover, skeletal muscle wasting was associated with increased therapy-induced toxicity and worse recurrence-free and overall survival [24,25,26]. A recently published study reported that the occurrence of major post-operative complications was associated with smaller total psoas muscle area and volume [27]. Whether the above-described body composition parameters are also associated with worse HRQoL before tumor resection and during the first year of disease has not yet been investigated.

The aims of the present study are (i) to describe patients’ body composition at diagnosis, (ii) to investigate associations between body composition at the time of diagnosis and HRQoL at time prior to tumor resection, and six and twelve months post-surgery, and (iii) to study associations between body composition and longitudinal HRQoL during the period between tumor resection and twelve months post-surgery in colorectal cancer patients. We hypothesize that unfavorable CT-based body composition at the time of diagnosis—in particular, high visceral fat area and low skeletal muscle mass (SMM) at colorectal cancer diagnosis—can be associated with impaired HRQoL.

## 2. Materials and Methods

### 2.1. Study Participants

Data from the prospective ColoCare Study (ClinicalTrials.gov identifier: NCT02328677) were used [28]. Eligible patients were newly diagnosed with a primary invasive colorectal cancer, 18 years or over, stage I–IV, German-speaking, and able to provide informed consent. Patients were recruited prior to tumor resection (baseline) at the Department of General, Visceral and Transplantation Surgery, University of Heidelberg, Germany, between 2010 and 2014. Participants were staged according to the American Joint Committee on Cancer (AJCC) system based on histopathologic findings. Colorectal cancer patients who underwent a multidetector-CT with quantification of visceral and subcutaneous fat area (VFA, SFA) and SMM and who fulfilled the European Organization for Research and Treatment of Cancer Core Quality of Life Questionnaire (EORTC QLQ-C30) prior to tumor resection, as well as six and twelve months post-surgery, were considered for analyses. Thus, a total of 138 patients were included in the analyses. The ColoCare Study was approved by the ethics committee of the Medical Faculty at the University of Heidelberg. All study participants provided written informed consent.

### 2.2. Data Collection

Details of medical and treatment factors were abstracted from patients’ medical records from the Heidelberg University Hospital. Self-reported questionnaires were used to collect sociodemographic data, alongside a set of multiple exposures among study participants as well as a variety of potential risk factors, including lifestyle characteristics.

### 2.3. Patient Reported Health-Related Quality of Life

As reported previously, cancer-specific HRQoL outcomes were measured by the patient-reported, validated core questionnaire QLQ-C30 [2], developed by the EORTC [29]. The EORTC QLQ-C30 includes one global health/quality of life (QoL) status, five functional scales (cognitive, emotional, physical, role, and social), three symptom scales (fatigue, nausea and vomiting, and pain), and six items reflecting symptoms and the financial impact of the disease [30]. All scales were transformed into a range from zero to 100 using recommended scoring methods. Higher scores of the global health/QoL status and functional scales indicate a better HRQoL. In contrast, higher symptom scores reflect a greater intensity of symptoms and thus a worse HRQoL [2,29]. For the purpose of this study, six scores and single items of the EORTC QLQ-C30 questionnaire were a priori selected (global health/QoL status, physical functioning, role and social functioning, fatigue, and pain).

### 2.4. CT-Defined Body Composition Parameters

**Figure 1:**
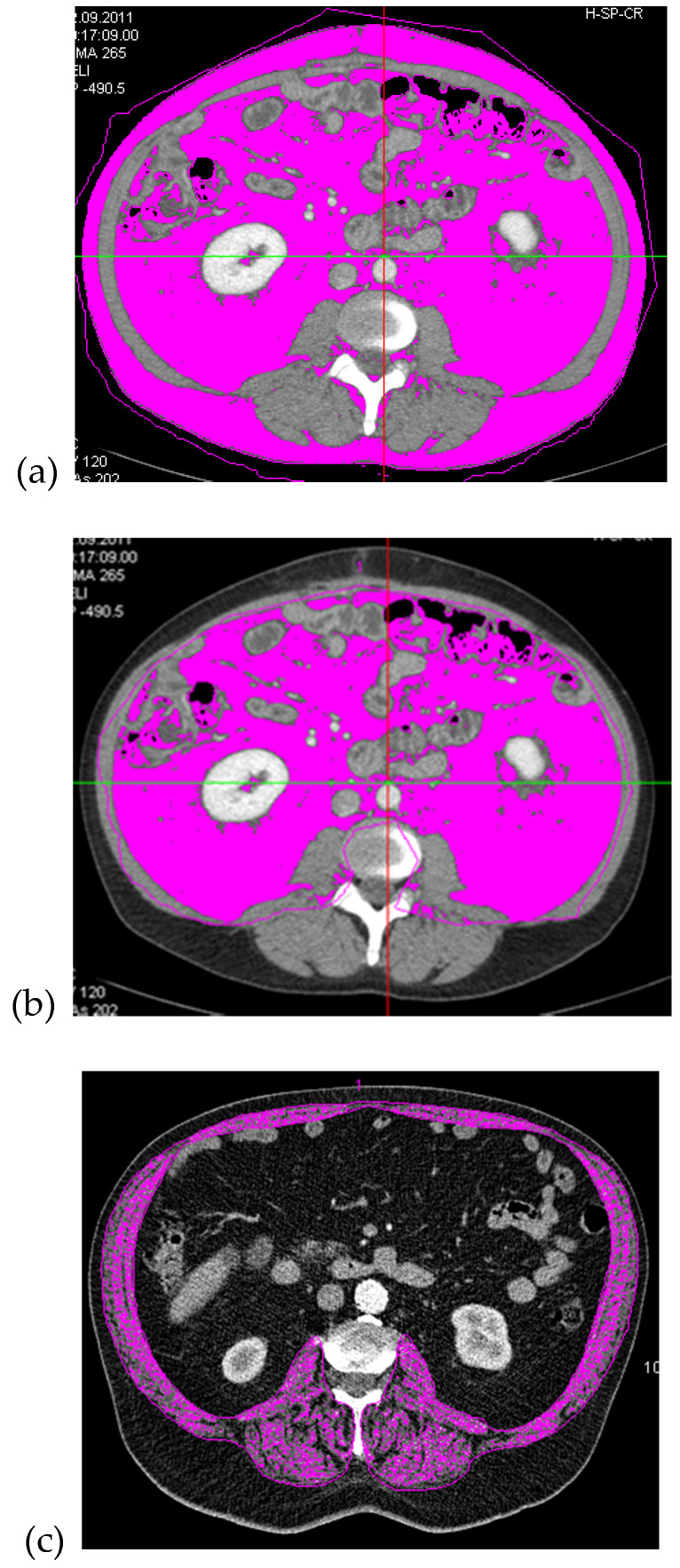
Example of a computed tomography (CT)-scan with the area-based, densitometric quantification of adipose tissue (threshold: −190 to −30 HU) measured at spinal level L3/4: regions of interest (ROI) containing total fat area (TFA) (**a**) and visceral fat area (VFA) (**b**); and an example of the densitometric quantification of muscle area, also measured at spinal level L 3/4 with an ROI containing the muscle tissue of the abdominal, dorsal and psoas muscles (threshold: 40 to 100 HU) (SMM, (**c**). Computed tomography (CT); Hounsfield units (HU); regions of interest (ROI); skeletal muscle mass (SMM); total fat area (TFA), visceral fat area (VFA).

CT scan based densitometric quantification has been described in detail elsewhere [18]. Briefly, area-based quantification of adipose tissue compartments was performed on the L3/4 spinal level (volumetric quantification of selected slice, divided by slice thickness) using a semiautomatic software tool (Syngo Volume tool, Siemens Healthcare, Munich, Berlin, Germany). Specific regions of interest (ROI) were manually determined: total fat area (TFA) (whole abdominal circumference, Figure 1a) and VFA (by defining the fascial plane of the abdominal muscle wall, Figure 1b). The limits of measurement for selecting the specific fat area were −190 HU (Hounsfield units) to −30 HU. SFA was calculated by subtracting VFA from TFA. On the same image slice, at the L3/4 spinal level, an ROI determining the SMM (sum of *M. psoas major*, *M. erector spinae*, *M. quadratus lumborum*, *M. latissimus dorsi*, *M. transversus abdominis*, *M. obliquus internus abdominis* and *externus abdominis*, and *M. rectus abdominis;* Figure 1c) was measured by limiting the attenuation threshold between 40 HU and 100 HU (excluding muscle lipid content; thresholds were based on visual controls which were conducted as a plausibility test within our study cohort) and normalized for height squared (see Figure 1) [13,18].

### 2.5. Statistical Analysis

Patients’ baseline characteristics are presented as the mean ± standard deviation or frequencies and concurrent percentages. Kruskal–Wallis tests, *t*-tests and a one-way analysis of variance (ANOVA) were applied for multiple group comparison. Spearman correlation coefficients were calculated to assess the correlations between body composition parameters and HRQoL scales. Concurrently, we used univariate regression models to determine the associations between body composition and HRQoL parameters.

To investigate the associations between body composition measurements (VFA, SFA and SMM) and longitudinal HRQoL, we used multivariate linear regression models adjusted for preselected potential confounders: (i) at the baseline time point models were adjusted for age at diagnosis (years), gender (female, male), BMI (kg/m^2^) at baseline, tumor stage (I to IV), tumor site (rectum, colon), and neoadjuvant treatment (yes, no), (ii) at the six month follow-up, instead of neoadjuvant, we used adjuvant treatment (yes, no) and adjusted for baseline HRQoL parameters and (iii) at the twelve month follow-up, we additionally adjusted for the six-month HRQoL levels. We performed four different multivariate linear models; first, we used VFA, SFA and SMM as covariates within one model, and then separately. In addition, we investigated multivariate linear models using BMI at baseline as a covariate, to show whether the signals using VFA, SFA and SMM were stronger than for BMI.

Finally, ordinal multivariate logistic regression models were used to assess the associations between body composition parameters and HRQoL changes. HRQoL changes were calculated by subtracting baseline scores from six-month follow-up scores and baseline scores from twelve-month follow-up scores. Differences were categorized into deterioration, clinically trivial change, and improvement based on the evidence-based guidelines for interpreting change scores for the EORTC QLQ-C30, with improvement as the lowest category [31]. Thus, an OR > 1 is interpreted as an “increased risk of deterioration”, which corresponds to the worst outcome of a specific HRQoL score. Models were adjusted in the same way as the linear models, except for six months, where we did not include baseline HRQoL as a covariate. At twelve months, we performed the analysis without baseline HRQoL scales. Furthermore, first, we included six-month HRQoL and second, excluded six-month HRQoL as a covariate. All tests were two-sided, *p*-values below 0.05 were considered significant. All analyses were performed using SAS 9.4 (SAS Institute, Cary, NC, USA).

## 3. Results

### 3.1. Participant Characteristics

In total, 387 eligible colorectal cancer patients were recruited between October 2010 and December 2014. A total of 38 patients died within the first year after enrolment. A total of 116 patients were excluded from the current analysis for having incomplete or no CT-based body composition quantification. Out of the retained 233 patients, 19 patients did not complete the EORTC QLQ-C30 parameters prior to surgery. Furthermore, 41 participants did not provide HRQoL information at six months and an additional 35 did not provide HRQoL information at twelve months post-surgery. Thus, the presented study included *n* = 138 colorectal patients with a mean age of 61.0 ± 11.5 years. A total of 28.3% were female. The majority had a history of rectum cancer (63%), while 37% were diagnosed with colon cancer. Non-participants had a higher mean age at diagnosis (64.3 ± 12.8) than participating colorectal cancer patients included in the analyses. Furthermore, compared to included patients, excluded patients were more likely to be female (differences >10%) and less often had rectum cancer (differences >10%). No other characteristics distinguished the included and non-included patients. Additional baseline patient characteristics are presented in Table 1.

### 3.2. Body Composition

VFA was significantly higher in men compared to women (*p* < 0.001; (mean VFA in male 206.4 cm^2^; 115.1 cm^2^ in female)). Furthermore, SMM was significantly higher in men compared to women (*p* < 0.01; (in females, SMM was 23.1 cm^2^/m^2^, compared to 27.8 cm^2^/m^2^ in male patients)). However, we did not observe significant differences in BMI between women and men. Moreover, increased age was positively correlated with VFA (r_VFA_ = 0.42, *p* < 0.01), and inversely correlated with SMM (r_SMM_ = −0.49, *p* < 0.01). Age did not correlate with SFA and BMI, respectively. Also, there were no significant differences in any body composition parameter across tumor sites and tumor stages, respectively. Patients who underwent neoadjuvant treatment had significantly lower BMIs at baseline compared to those who did not receive treatment before surgery (*p* = 0.03).

### 3.3. Associations of Adipose and Muscle Tissue with Health-Related Quality of Life

In univariate analyses, at baseline and six months post-surgery, SMM was positively correlated with physical functioning (r_SMM_ = 0.19, *p* = 0.02 and r_SMM_ = 0.18, *p* = 0.04, respectively). At the six-month follow-up, VFA was inversely correlated with social functioning (r_VFA_ = −0.17, *p* = 0.04) and SMM was inversely correlated with pain scores (r_SMM_ = −0.19, *p* = 0.03). At twelve months post-surgery, we observed an inverse correlation between VFA and physical functioning (r_VFA_ = −0.18, *p* = 0.03), see Table 2.

After adjusting for potential confounders, we did not observe any associations between CT-quantified body composition parameters and any HRQoL scale at baseline. At the six-month follow-up, after adjustment higher VFA continued to be associated with worse social functioning (β = −0.08, *p* = 0.01). Moreover, at the six- and twelve-month follow-up, VFA was associated with higher pain scores (β = 0.06, *p* = 0.04 and β = 0.07, *p* = 0.01). Additionally, at the twelve-month time point, higher SMM was associated with increased pain (β = 1.03, *p* < 0.01), see Table 3.

In our independent analysis, using BMI at baseline as an independent covariate, we did not observe any associations between BMI and HRQoL scales. In our multivariate ordinal logistic regression models, all results were comparable to the initial models using linear regressions; see Table 4.

## 4. Discussion

We investigated the associations of CT-based body composition parameters, in particular visceral fat area (VFA), subcutaneous fat area (SFA) and skeletal muscle mass (SMM) at diagnosis with longitudinal HRQoL in colorectal cancer patients, describing the first year of disease. To our knowledge, this is the first study investigating these associations in a prospective, longitudinal manner. The multivariate results reported here provide a portrayal of associations between unfavorable body composition factors and the global health/QoL status, social functioning, and clinical symptoms affecting colorectal cancer patients after surgery [32].

A higher visceral fat amount at diagnosis was associated with higher pain scores at six and twelve months after tumor resection. Our results support previous research data showing that visceral obesity at colorectal cancer diagnosis was associated with a higher rate of post-operative complications, such as wound infection [19] and increased dose-limiting toxicity [24], and that post-operative complications were associated with worse HRQoL [33]. At six months post-surgery, greater amounts of visceral fat were associated with deteriorated social functioning. Additionally, in our cohort, pain was highly correlated with worse social functioning (data not shown). Worse pain scores and associated visceral obesity may limit patients’ ability to participate in valued activities such as hobbies or physical activity [34], which, in turn, may affect social QoL. Our data show that the amount of VFA was higher in male and older patients, which is in line with other cancer cohorts [35].

Similar findings regarding the importance of body composition in cancer outcomes have also been shown in other cancer studies. Recently, Aleixo et al. reported that poor body composition with high visceral adipose tissue and lower muscle tissue and density is associated with physical function which, in turn, may affect treatment tolerability and other cancer outcomes among women with early stage breast cancer [36]. Moreover, Sheean and colleagues showed that obese women with estrogen receptor-positive metastatic breast cancer had significantly higher levels of abdominal obesity and serum biomarkers of inflammation, with a lower quality of life compared to women without obesity [37].

Study results from Swisher et al. and Gudmundsson et al. in breast cancer and different types of cancer survivors, respectively, underlined the importance of exercise, fitness and dietary counseling, which led to a loss of body fat and an improved quality of life in cancer patients [38,39].

Interestingly, and in contrast to our hypotheses, patients with higher SMM at diagnosis suffered more from pain one year after tumor resection. One explanation for this observation might be that, during the first year of disease, patients experienced a rapid loss of muscle mass, (cancer-associated sarcopenia [40]), which might occur especially in patients with more muscle mass at diagnosis.

The findings outlined above suggest that visceral obesity at the time of diagnosis may unfavorably and selectively affect patients’ HRQoL during the first year after tumor resection for colorectal cancers. Moreover, these findings may have implications for healthcare practitioners who work with male and older colorectal cancer patients. In this context, the prevention of an unfavorable body composition, e.g., via nutritional intervention and physical exercise, particularly resistance training, may potentially improve patients’ HRQoL.

The strengths of this study are its longitudinal design with repeated measurements of HRQoL during the first year after tumor resection. To date, this is the first prospective investigation into the associations between CT-based body composition and longitudinal (short-term) HRQoL in colorectal cancer patients. Previous studies reported that colorectal cancer survivors experience clinically relevant detriments in HRQoL even ten years after diagnosis [41,42] and early identification of patient characteristics that determine patients’ short-term HRQoL are needed. Our study results show that colorectal cancer patients with unfavorable body composition at diagnosis experience lower HRQoL during the first year of disease and this provides important implications for clinical practice. These patients may have a particular need for interventions to reduce visceral fat. Another important strength of our study is the use of CT images to define body composition. CT-based body composition analysis enables us to accurately and precisely determine fat and muscle tissue at colorectal cancer diagnosis. Moreover, CT imaging is established as a routinely diagnostic procedure in cancer patients and no further measurements of body composition are needed, which means no further testing exposure and no additional burdens for patients. Furthermore, we used a standardized and well-established cancer-specific HRQoL instrument, which allows for a comparison to be made with other cohorts and research.

This study is subject to the following limitations: we only included patients with existing CT scans in this study. Therefore, patients without CT scans (patients with magnetic resonance imaging or low-stage patients who did not receive any routine imaging) are underrepresented. We did not perform any further CT scans for study purposes due to radiation protection. We measured muscle tissue using a threshold limit of 40 to 100 HU to selectively obtain the dense and functional relevant parts of muscle tissue without fatty infiltration. In the literature, a wider threshold (−29 to 150 HU) is given to measure the total muscle tissue [12,43]; thus, our results on muscle tissue are limited in comparison to other studies. However, a within-cohort comparison is still given. Furthermore, although our study is a large prospective investigation, some results might suffer from limited statistical power. Since no correction for multiple testing was performed on our data, we cannot rule out that some of the results are false positives. Moreover, possible selection bias can arise, as we cannot rule out that patients who experienced worse HRQoL did not participate or complete questionnaires at follow-up time points and thus are underrepresented in our study. Another limitation of our study is the lack of follow-up CT scans and thus a lack of the availability of body composition measures one year after diagnosis in order to describe potential changes in adipose tissue and muscle mass over time.

## 5. Conclusions

Patients with high amounts of visceral fat at diagnosis exhibited worse scores for social functioning and deteriorated pain after surgery, independent of treatment. Additionally, patients with higher skeletal muscle mass at diagnosis suffered more from more pain one year after surgery. This might reflect a rapid loss of muscle mass (cancer-associated sarcopenia) occurring in and especially affecting patients with more muscle mass at diagnosis. Our results suggest that intervention strategies targeting visceral fat and muscle mass might improve the health-related quality of life in colorectal cancer patients during the first year after surgery.

## Figures and Tables

**Table 1:** Baseline patient characteristics.

	*n* = 138 Patients Included in Analyses
**Age** years (mean ± SD)	61.0 ± 11.5
**Gender***n* (%)	
Female	39 (28.3)
Male	99 (71.7)
**Tumor site***n* (%)	
Colon	51 (37.0)
Rectum	87 (63.0)
**Tumor stage***n* (%)	
I	28 (20.3)
II	52 (37.7)
III	37 (26.8)
IV	21 (15.2)
**Neoadjuvant treatment***n* (%)	
No	85 (61.6)
Yes	53 (38.4)
**Adjuvant treatment***n* (%)	
No	69 (50.0)
Yes	69 (50.0)
**BMI at baseline** kg/m^2^ (mean ± SD)	
Female	25.9 ± 4.9
Male	27.6 ± 3.8
**VFA** cm^2^ (mean ± SD)	
Female	115.1 ± 76.3
Male	206.4 ± 102.9
**SFA** cm^2^ (mean ± SD)	
Female	225.8 ± 109.4
Male	225.8 ± 103.8
**SMM** cm^2^/m^2^ (mean ± SD)	
Female	23.1 ± 7.1
Male	27.8 ± 10.3

Standard deviation (SD), body mass index (BMI), visceral fat area (VFA), subcutaneous fat area (SFA), skeletal muscle mass (SMM).

**Table 2:** Correlation of VFA, SFA, SMM, and BMI with HRQoL scales.

	Baseline (Prior to Surgery)				6 Months Post-Surgery				12 Months Post-Surgery			
	VFA	SFA	SMM	BMI	VFA	SFA	SMM	BMI	VFA	SFA	SMM	BMI
**Global health/QoL**												
r	−0.002	−0.05	0.03	−0.08	−0.14	0.02	0.04	−0.08	−0.1	0.02	−0.08	−0.07
*p*-value	0.98	0.54	0.68	0.33	0.09	0.8	0.66	0.34	0.25	0.83	0.36	0.42
**Physical functioning**												
r	−0.06	0.03	*0.19*	−0.04	−0.14	−0.01	*0.18*	−0.05	*−0.18*	−0.01	0.13	−0.08
*p*-value	0.51	0.69	*0.02*	0.65	0.1	0.91	*0.04*	0.58	*0.03*	0.9	0.14	0.38
**Role functioning**												
r	−0.1	−0.04	0.07	−0.07	−0.09	0.04	0.04	−0.02	−0.1	−0.01	−0.06	−0.06
*p*-value	0.25	0.66	0.42	0.44	0.31	0.65	0.63	0.78	0.25	0.92	0.45	0.49
**Social functioning**												
r	0.11	0.11	−0.06	0.08	*−0.17*	0.11	−0.11	−0.01	−0.14	−0.005	−0.05	−0.07
*p*-value	0.18	0.2	0.52	0.38	*0.04*	0.19	0.2	0.94	0.09	0.95	0.56	0.42
**Fatigue**												
r	0.004	−0.03	−0.11	−0.03	0.13	−0.02	−0.1	0.003	0.1	−0.03	−0.03	0.04
*p*-value	0.96	0.72	0.2	0.7	0.13	0.83	0.22	0.96	0.24	0.72	0.74	0.68
**Pain**												
r	−0.06	−0.07	−0.08	−0.06	0.12	0.03	*−0.19*	0.07	0.1	0.15	0.1	0.12
*p*-value	0.5	0.4	0.36	0.48	0.16	0.7	*0.03*	0.44	0.24	0.08	0.24	0.17

Univariate analysis using Spearman correlation coefficients. Spearman correlation coefficients and *p*-values in *italics* indicate a statistically significant correlation between CT-based body composition parameters and HRQoL scales. Health-related quality of life (HRQoL), quality of life (QoL), body mass index (BMI), visceral fat area (VFA), subcutaneous fat area (SFA), skeletal muscle mass (SMM), Spearman correlation coefficient (r).

**Table 3:** Multivariate analyses of associations of VFA, SFA, and SMM with HRQoL scales.

	Baseline (Prior to Surgery)			6 Months Post-Surgery			12 Months Post-Surgery		
	VFA	SFA	SMM	VFA	SFA	SMM	VFA	SFA	SMM
**Global health/QoL**									
β	0.005	−0.02	−0.19	−0.03	0.01	−0.03	−0.01	0.003	−0.02
*p*-value	0.84	0.47	0.5	0.23	0.45	0.89	0.72	0.85	0.93
**Physical functioning**									
β	−0.02	0.02	0.18	−0.03	0.001	0.18	−0.02	0.01	−0.05
*p*-value	0.49	0.32	0.44	0.17	0.95	0.41	0.17	0.28	0.75
**Role functioning**									
β	−0.005	−0.005	−0.27	−0.04	0.02	0.1	−0.02	0.005	−0.4
*p*-value	0.89	0.87	0.47	0.29	0.55	0.77	0.48	0.83	0.18
**Social functioning**									
β	0.04	0.02	0.12	*−0.08*	0.02	−0.33	0.002	−0.03	−0.19
*p*-value	0.26	0.56	0.72	*0.01*	0.45	0.29	0.95	0.26	0.51
**Fatigue**									
β	0.02	−0.02	−0.15	0.05	−0.01	−0.01	0.02	−0.02	−0.02
*p*-value	0.56	0.44	0.64	0.09	0.56	0.97	0.31	0.32	0.92
**Pain**									
β	−0.03	−0.02	−0.32	*0.06*	−0.002	−0.39	*0.07*	0.02	*1.03*
*p*-value	0.37	0.42	0.37	*0.04*	0.92	0.2	*0.01*	0.42	*0.0006*

Multivariate analysis using linear regression models. *Italics* indicate statistically significant associations between CT-based body composition parameters and HRQoL scales. For baseline analyses, models were adjusted for age, gender, tumor stage, tumor site and neoadjuvant treatment. For six-month follow-up analyses, models were adjusted for age, gender, tumor stage, tumor site, adjuvant treatment and baseline HRQoL scores. For twelve-month follow-up analyses, models were adjusted for age, gender, tumor stage, tumor site, adjuvant treatment, and baseline and six-month HRQoL scores. Health-related quality of life (HRQoL), quality of life (QoL), visceral fat area (VFA), subcutaneous fat area (SFA), skeletal muscle mass (SMM), estimate (β).

**Table 4:** Multivariate analyses of associations of VFA, SFA, and SMM with HRQoL scales.

	6 Months Post-Surgery			12 Months Post-Surgery		
	VFA	SFA	SMM	VFA	SFA	SMM
**Global health/QoL**						
OR (CI)	1.05 (0.86–1.28)	0.95 (0.78–1.11)	1.08 (0.70–1.69)	1.05 (0.86–1.35)	0.86 (0.70–1.00)	0.80 (0.51–1.24)
*p*-value	0.57	0.49	0.72	0.62	0.09	0.32
**Physical functioning**						
OR (CI)	1.16 (0.95–1.49)	1.05 (0.86–1.22)	1.15 (0.74–1.81)	1.05 (0.82–1.28)	1.05 (0.86–1.28)	1.13 (0.72–1.77)
*p*-value	0.13	0.76	0.55	0.78	0.66	0.59
**Role functioning**						
OR (CI)	1.11 (0.91–1.05)	0.95 (0.82–1.16)	1.16 (0.74–1.82)	1.11 (0.91–1.35)	1.00 (0.82–1.22)	1.23 (0.78–1.93)
*p*-value	0.28	0.77	0.52	0.39	0.95	0.37
**Social functioning**						
OR (CI)	*1.35 (1.05–1.65)*	1.00 (0.82–1.22)	1.24 (0.79–1.99)	1.16 (0.95–1.42)	1.00 (0.82–1.22)	0.99 (0.64–1.54)
*p*-value	*0.01*	0.95	0.33	0.14	0.99	0.97
**Fatigue**						
OR (CI)	1.05 (0.86–1.28)	0.95 (0.78–1.16)	1.04 (0.67–1.63)	1.16 (0.91–1.42)	0.91 (0.74–1.11)	1.06 (0.68–1.66)
*p*-value	0.61	0.61	0.85	0.24	0.25	0.8
**Pain**						
OR (CI)	*1.28 (1.05–1.65)*	1.11 (0.91–1.28)	0.83 (0.54–1.29)	*1.35 (1.11–1.65)*	1.05 (0.91–1.28)	*1.66 (1.06–2.67)*
*p*-value	*0.02*	0.4	0.42	*<0.01*	0.45	*0.03*

Multivariate analysis using ordinal logistic regression models. *Italics* indicate statistically significant associations between CT-based body composition parameters and HRQoL scales. Odds ratios and corresponding confidence intervals were scaled to reflect a change of 10 units (cm^2^/m^2^) for height-normalized values (SMM), and a change of 50 units (cm^2^) for adipose tissue area L3/4 (VFA and SFA). For six-month follow-up analyses, comparing changes (improvement, clinically trivial changes, or deterioration) in HRQoL scales between six months post-surgery and baseline, models were adjusted for age, gender, tumor stage, tumor site, and adjuvant treatment. For twelve month follow-up analyses, comparing changes (improvement, clinically trivial changes, or deterioration) in HRQoL scales between twelve months post-surgery and baseline, models were adjusted for age, gender, tumor stage, tumor site, adjuvant treatment, and six-month HRQoL scores. Health-related quality of life (HRQoL), quality of life (QoL), visceral fat area (VFA), subcutaneous fat area (SFA), skeletal muscle mass (SMM), odds ratio (OR), confidence interval (CI).

## Abbreviations

ANOVA: Analysis Of Variance; BMI: Body Mass Index; CT: Computed Tomography; EORTC QLQ: European Organization for Research and Treatment of Cancer Core Quality of Life Questionnaire; ICD: International Statistical Classification of Disease and Related Health Problems; OR: Odds Ratio; HRQoL: Health-related quality of life; HU: Hounsfield Units; QoL: Quality of Life; ROI: Regions of Interest; SAS: Statistical Analysis System; SFA: Subcutaneous Fat Area; SD: Standard Deviation; SMM: Skeletal Muscle Mass; VFA: Visceral Fat Area.
